# Electrolyte imbalance in a patient with weight loss: what is the cause?

**DOI:** 10.1007/s40620-025-02259-9

**Published:** 2025-03-26

**Authors:** Ophir Eyal, Yael Golomb

**Affiliations:** Department of Nephrology and Hypertension, Division of Internal Medicine, Hadassah Medical Center, Kiryat Hadassah, POB 12000, 91120 Jerusalem, Israel

**Keywords:** Hypokalemia, Hypophosphatemia, Fanconi syndrome, Sjogren’s Disease

A woman in her mid-50s presented to the emergency department due to progressive proximal myalgia and weakness lasting for about one month. The weakness increased to the point of difficulty in sitting up in bed, prompting her to seek medical attention. Of note, 7 months prior to admission she started therapy with liraglutide for obesity, resulting in a decrease in appetite and a consequent gradual weight loss reaching 20 kg at the time of presentation. She denied vomiting or diarrhea.

Additional history included ring gastroplasty 25 years previously. Twenty-five months prior to her current presentation, lumbar laminectomy was performed. This was complicated by surgical wound infection requiring prolonged intravenous antibiotic treatment, for which a peripherally inserted central line was placed. This, in turn, was complicated by thrombus development around the line, leading to long-term anticoagulant therapy. She had also previously undergone evaluation for arthralgias. While serologic workup was positive for antinuclear antibodies (ANA) and anti-SSA, the diagnosis was fibromyalgia.

Home medications at presentation included apixaban 2.5 mg twice daily, omeprazole 20 mg once daily, liraglutide 0.6 mg once daily, bilastine 20 mg once daily and pregabalin 150 mg once daily.

Upon presentation, her BMI was 34.3; her vital signs were normal and cardiopulmonary and abdominal examinations were without significant findings. Initial bloodwork on admission showed hypokalemia 1.9 meq/L (range 3.5–5.1 meq/L) and hypophosphatemia 2.0 mg% (range 2.4–5.1 mg%). Previous tests, performed 10 months prior, demonstrated no such abnormalities.

Calculations of the urine potassium/creatinine ratio and Tm_PO4_/GFR were consistent with renal losses of potassium and phosphate, respectively (see Table 1 for all laboratory results). As this suggested Fanconi syndrome, additional tests of proximal tubule function were performed, which showed glucosuria in a non-diabetic normoglycemic patient not treated with an SGLT-2 inhibitor, hypouricemia with inappropriate uricosuria and normal anion gap (hyperchloremic) metabolic acidosis with adequate respiratory compensation. The high urinary pH of 7.3 in the absence of bicarbonate treatment was suggestive of a distal renal tubular acidosis. Testing for aminoaciduria was not available.

**Table 1:** Laboratory data

Variable	Upon admission	Reference range
Blood		
Sodium (mmol/liter)	139	136–145
Chloride (mmol/liter)	112	98–107
Potassium (mmol/liter)	1.9	3.5–5.1
Calcium (mg/dl)	9.3	8.3–10.6
Phosphorus (mg/dl)	2	2.4–5.1
Uric acid (mg/dl)	2.3	3.1–7.8
Glucose (mg/dl)	116	74–106
pH	7.343	7.32–7.45
HCO_3_^−^ (mmol/liter)	17.3	18–24
pCO_2_ (mmHg)	32.5	41–51
Albumin (g/dl)	4.4	3.2–4.8
Creatinine (mg/dl)	0.88	0.55–1.02
Urine		
Creatinine (mg/dl)	68.2	600–1800
Sodium (mmol/liter)	27	40–220
Potassium (mmol/liter)	21	< 15 mmol/liter*
Potassium/Creatinine	30.8	< 1.5*
Phosphorous (mg/dl)	15.9	
Fractional Excretion of filtered P (%)	10.3	< 5%**
Tm_PO4_/GFR	0.8	0.88–1.42
Uric acid (mg/dl)	26.9	3.5–7.2
Fractional Excretion of Uric Acid (%)	15.9	5–9%***
Glucose (mg/dl)	6	0–0.5
pH	7.3	4.5–8

*In hypokalemic patients with non-renal potassium loss

**In hypophosphatemic patients with non-renal phosphate loss

***In euvolemic patients

**What is the differential diagnosis?**

**What is the most likely etiology in this patient?**

**How should this patient be best treated?**

## Case solution

The patient presented with myalgia and muscle weakness, explained by severe hypokalemia and hypophosphatemia. While these could be attributed to decreased intake under liraglutide treatment, dietary history and weight loss rate did not support severe malnutrition.

Gastrointestinal losses were ruled out per history, increasing suspicion of urinary losses. While the gold standard for assessing urinary loss of electrolytes is a 24-h urine collection, this is often not readily  feasible, necessitating tests on a spot urine sample. In this case, the urinary potassium/creatinine ratio was 30.8 mEq/g, and the fractional excretion of phosphate was 10.3% with Tm_PO4_/GFR of 0.8, signifying inappropriate urinary losses.

The proximal tubule is the main reabsorption site for both electrolytes, raising concern for general dysfunction of this segment, i.e. Fanconi syndrome. The full syndrome also includes loss of glucose, uric acid, bicarbonate (i.e. proximal renal tubular acidosis) and amino acids. Our patient had urinary losses of glucose and uric acid, while aminoaciduria was not tested for.

The etiologies of Fanconi syndrome are presented in Table 2. In our patient, age at onset and lack of family history ruled out genetic etiologies. There was no history of exposure to heavy metals or relevant drugs.

**Table 2:** Major causes of Fanconi Syndrome [1–3]

Inherited causes	Acquired causes
Cystinosis Galactosemia Hereditary fructose intolerance Tyrosinemia Wilson disease Lowe syndrome Dent disease Glycogenosis Mitochondrial cytopathies Idiopathic	Drugs: Cisplatin, Ifosfamide, Tenofovir, Cidofovir, Adefovir, Didanosine, Gentamicin, Azathioprine, Valproic acid, Suramin, Streptozocin, Ranitidine Heavy metals: lead, cadmium Dysproteinemias: multiple myeloma, light chain proteinuria, amyloidosis Sjögren’s syndrome Chinese herbal medicine: aristolochic acid Toluene: Glue sniffing Nephrotic syndrome Renal transplantation Acute tubular necrosis

A major cause of Fanconi syndrome in the adult population is dysproteinemia in  multiple myeloma or monoclonal gammopathy of renal significance (MGRS). This was ruled out by the absence of paraprotein in serum or urine and a normal free light chains ratio.

Sjögren’s Syndrome is a cause of Fanconi syndrome and distal renal tubular acidosis. Focused history revealed minor complaints of dry mouth and eyes. Serology showed positive ANA with very high anti-SSA titer, mildly positive anti-SSB, normal complement levels, elevated rheumatoid factor at 64 (normal < 14) and negative cryoglobulins.

Over the subsequent months, plasma creatinine remained stable at 0.7 mg/dL with proteinuria around 700 mg/day and undetectable urine albumin, consistent with tubular proteinuria.

The kidney is a significant target organ of Sjögren’s Syndrome. Alongside electrolyte abnormalities and nephrolithiasis, renal involvement typically includes tubulointerstitial nephritis.

The electrolyte abnormalities were controlled with oral supplements. Immunosuppression for Sjögren’s Syndrome-associated nephritis includes prednisone and possibly mycophenolate mofetil or cyclophosphamide. On the contrary, treatment for tubulopathy alone is ill defined [4, 5]. Steroid treatment to prevent renal function deterioration was discussed; weighing the scant supporting evidence and metabolic side effects, the patient preferred surveillance and, 18 months since presentation, the patient is feeling well, with normal laboratory electrolyte findings.﻿

## Data Availability

All the relevant data are already included in the manuscript.
